# Research on Low-Velocity Impact Response of Novel Short-Fiber-Reinforced Composite Laminates

**DOI:** 10.3390/polym15040840

**Published:** 2023-02-08

**Authors:** Yinyuan Huang, Felix Thompson EShun, Junfeng Hu, Xutong Zhang, Jianping Zhao, Siqi Zhang, Rui Qian, Zhou Chen, Dingding Chen

**Affiliations:** 1 School of Mechanical and Power Engineering, Nanjing Tech University, Nanjing 211800, China; 2 Jiangsu Bi-gold New Material Stock Co., Ltd., Zhenjiang 212499, China; 3 College of Aerospace Science and Engineering, National University of Defense Technology, Changsha 410073, China

**Keywords:** UACS laminates, low-velocity impact, damage behavior, finite element simulation

## Abstract

Short-fiber-reinforced polymers (SFRPs) based on unidirectionally arrayed chopped strands (UACSs) have excellent formability and outstanding mechanical response. The low-velocity impact response, such as the delamination, damage tolerance and energy absorption of UACS composites, are essential to guarantee the stability and safety of composite components in service. The current study investigates the low-velocity impact response of continuous carbon-fiber-reinforced polymer (CFRP) and UACS laminates with vertical slits under drop-weight impact with various impact energies (4, 7 and 11 J). The in-plane size of the studied samples is 100 mm × 100 mm, and the stacking sequence is [0/90]_4s_. The time–history curves of load and energy are examined during low-velocity impact experiments, as well as the nonvisible damages are obtained by ultrasound C-scan imaging technique. A user-defined subroutine VUMAT, including the Johnson–Cook material and failure model, which is used to simulate the elastic–plastic property of the slits filled with resin, is coded in ABAQUS/Explicit. According to C-scan inspections of the impact-damaged laminates, UACS specimens show more severe delamination as impact energy increases. The damaged area of continuous CFRP laminates under impact energy of 11 J is 311 mm^2^, while that of UACS laminates is 1230 mm^2^. The slits have a negative effect on the load-bearing capacity but increase the energy absorption of UACS laminates by approximately 80% compared to the continuous CFRP laminates at 7 J. According to the variables of different damage modes in numerical simulation, cracks appear at the slits and then expand along the direction perpendicular to the slits, leading to the fracture of fiber. Nevertheless, as the damage expands to the slits, the delamination confines the damage propagation. The existence of slits could guide the path of damage propagation.

## 1. Introduction

Due to their high specific stiffness and strength, fiber composites have been used in many fields over recent years [1]. Among them, carbon-fiber-reinforced polymer composite (CFRP) materials, as an advanced composite material, have sets of benefits such as their low thermal expansion coefficient, lightweight, high specific strength and high modulus. They are extensively used in the aerospace industry, wind power industry, automobile industry, structural reinforcement engineering and other fields [2]. Traditional continuous CFRP composites have certain limitations when they are used to form components with complex shapes. In the curing process, uneven fiber and resin distribution easily occur. The existence of this kind of defect will inevitably produce a complex stress distribution inside the material, which will affect the structure’s safety performance. As a result, SFRP with exceptional fiber fluidity has been the subject of extensive research since the 1960s. For example, sheet molding compound (SMC) has been developed by leaps and bounds due to its excellent fiber fluidity and lower cost [3,4]. The molding processes can achieve proper formation; however, they are unable to regulate the microstructure of short fiber distribution and result in poor mechanical properties [5]. Taketa et al. [6] introduced UACS in which continuous CFRP prepreg is chopped into regular discontinuous distribution to further improve the equilibrium relationship between mechanical property and fiber flowability. Similarly, UACSs and SMCs are all stacked from multiple sheets. However, the primary distinction between UACSs and SMCs is the high structural regularity. When fibers are arranged in a regular pattern, UACSs exhibit excellent stiffness, which is comparable to continuous CFRPs, a strength that is much greater than SMCs, and high formability that is similar to SMCs. Hang Li et al. [7] also suggested new designs of UACSs by incorporating discontinuous angled slits, and their results showed better strength and flowability compared to the conventional UACS laminates.

Due to the fact that low-velocity impact is one of the major potential hazards for laminates, and UACS laminates are now being used in composite structures primarily for their strength and damage tolerance, an impact test on them is required to understand their impact behavior. Aircraft are concerned about low-velocity impact damage during taxiing, landing, and maintenance. Low-velocity impact damage to the wing skin can result from tool drops during maintenance or small pieces of ground debris hitting the wing skin when taxiing. The kind of instrument or object that strikes the structures will determine the type of impact damage that occurs. It could have a smooth, soft edge or a sharp edge. Due to accidents that occur during the manufacturing or maintenance processes, the components could lose more than half of their strength when confronted with low-velocity impact issues [8]. Conventional nondestructive testing methods are unable to find surface flaws or damages that are scarcely perceptible, as is typical in low-velocity impacts on composites. Numerous factors, including impact velocity and energy, projectile/impactor shape, stacking sequence, and boundary conditions, affect impact behavior and damage [9]. Damage modes for composite-fiber-reinforced laminates include matrix cracking, fiber breaking, and delamination. Under impact loading, these different damage modes are exhibited collectively [10]. Since most composites are fragile, plastic deformation is not the cause of the damage. Low-velocity impacts are characterized as incidents that can happen at speeds between 1 and 10 m/s based on the stiffness, mass and material qualities of the impactor [11]. A drop-weight impact testing device can simulate a variety of actual impact situations and gather thorough performance data [12].

The low-velocity impact response of composite laminates has been studied in several pieces of research using experimental observation [13], theoretical analysis [14,15,16,17,18,19] and numerical modeling. Numerical modeling is thought to be a useful tool to obtain precise damage propagation and is also ideal for parametrical research, considering the high cost of impact experiments. The progressive damage model (PDM), considering failure initiation and stiffness degradation, is the most common among the numerical theoretical models. When the failure criterion is reached in some area of the composite, the damage begins to occur, and visible or invisible cracks begin to expand, resulting in stiffness degradation and gradually reducing load capacity. Due to the correlation with mechanical parameters, the method based on fracture energy is widely used to predict the progressive damage behavior of composites, such as the equivalent displacement method, which has been well verified by a large number of experimental results [20,21,22]. In the main damage types that occur in laminates, delamination is viewed as the crucial one because it might drastically decrease the compression strength, stability and integrity of composites [23]. Considering the essentiality of this failure mechanism, researchers have used two basic techniques to anticipate delamination in laminates: the virtual crack closure technique (VCCT) [24] and the cohesive zone model (CZM) [25]. Nevertheless, the VCCT cannot anticipate fracture initiation and requires the use of an adaptive mesh reconstruction method in the delamination front. On the other hand, the CZM utilizes criteria based on strength and fracture energy to forecast damage beginning and propagation.

The interactive mixed mode serves as the foundation for the traction–separation law of cohesive elements for simulating delamination beginning and propagation. It makes sense when the through-thickness normal stress is under tension, but the delamination inhibition effect, which is highlighted by Hou et al. [26], is neglected when the normal stress is under compression. Through-thickness compression stress should be included in CZM because it might significantly increase the interfacial shear strength. In order to investigate the impact of compressive stress on the evolution of mode II damage, Li et al. [27] suggested a contact failure model to study the maximum compression stress on mode II damage propagation. This model used a separately derived parameter to link the rise in interlaminar strength properties and mode II key crack energy to the compression stress. In the quasi-static delamination modeling of composite structures, Zhang et al. [28,29] introduced an interfacial model to appreciate the interaction-force-generated friction impact on shear-induced mode II damage. In order to forecast low-speed impact damage in composite materials utilizing a cohesion contact based on surfaces and a quasi-static stress model, Zhang and Zhang [29] devised a numerically effective approach. By including interface friction in the shear strength directions, this model studied the impact of compressive interlaminar stress on deformation.

Based on the literature, there are few reports about research on UACS laminates under low-speed impact; hence, the goal of the current investigation is to determine its damage response and explore the influence of slits on the impact performance of UACS laminates with vertical slits. The damage mechanisms underlying this topic are of importance for the application of UACS composites. This triggered the analysis of the experimental and numerical study of the influence of vertical slits on the impact behavior of UACS laminates in the present work. A series of drop-weight impact experiments are conducted on the UACS composites under different impact energies. The impact response curves are analyzed, and the internal damage is examined with C-scan inspection. Parallelly, in order to model the progressive damage process of UACS composite structures, a 3D finite element simulation model is made in ABAQUS/Explicit, and the progressive damage model (PDM) is suggested and encoded in the user-specified subroutine (VUMAT), which incorporates the Hashin criterion and a computation technique for equivalent movement in the damage propagation model. To describe the effect of delamination occurring at the slits on the damage evolution, interface cohesive pieces with the nonlinear traction–separation law are inserted among plies. Finally, a discussion on the UACS laminates with vertical slits is performed for impact performance in damage tolerance and energy absorption.

## 2. Materials and Experimental Setup

### 2.1. Materials

**Figure 1:**
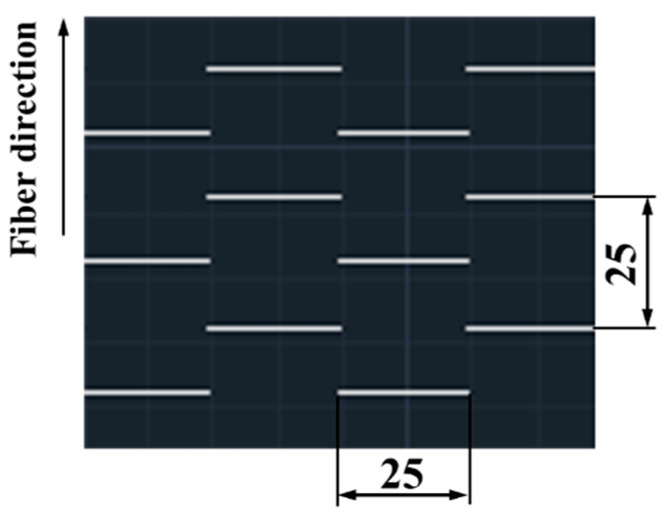
Unidirectional CFRP prepreg with vertical, chopped slits.

The composite materials used in this research were UIN12500 (T800) (Guangwei, Weihai, China), a commercial continuous CFRP prepreg of 0.15 mm thickness lamina and 60% fiber volume fraction. A quasi-isotropic lay-up sequence of [0/90]_4S_ was tested, and all the composite laminates were fabricated using 16 layers of the UACS laminates. The laminate was placed into a hot press machine at a pressure of 0.6 MPa and a compression temperature of 130 °C, according to the prepreg manufacturer’s supplied curing curve. The specimens were cut precisely using a water-jet cutting machine, and the vertical slits on the UACS prepreg were made by a commercial cutting tool. The final slit had nominal dimensions of 25 mm × 0.2 mm. Each of the UACS laminates had vertical slits, as illustrated in Figure 1. The specimen’s standard dimensions were kept at 100 mm in length and 100 mm in width. The laminates had an average thickness of 1.99 mm.

### 2.2. Experimental Procedure

**Figure 2:**
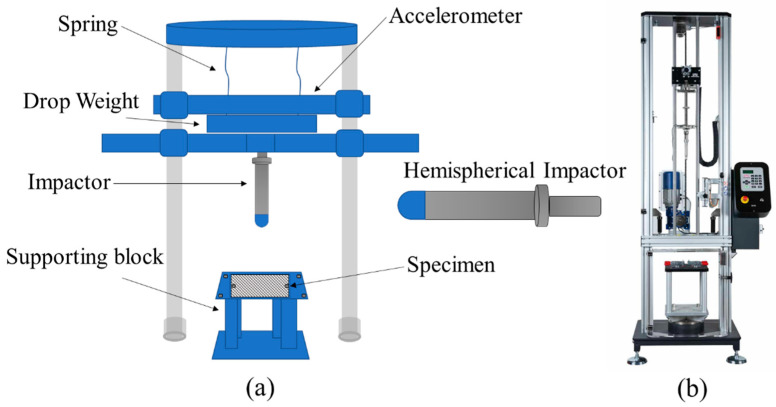
Impact setup on UACS laminate using the hemispherical impactor. (**a**) Schematic diagram. (**b**) Instron CEAST 9350 Drop Tower Impact System 3. Finite element modeling.

The cross-ply UACS composite materials were put through low-velocity impact tests for various impact energies of 4 J, 7 J, and 11 J at room temperature. Figure 2a shows the impact test setup and the impactor that was employed in this study. The specimen was fastened by a clamping steel plate with a 75 mm diameter hole in the center, and the top surface’s edges were tightened after synthetic rubber pads were positioned in the gap [30]. Samples from the UACS laminates were evaluated under impact loading using an impactor with a hemispherical shape with a 16 mm diameter. The drop-weight mass of the hemispherical impactor was 5.265 kg. As depicted in Figure 2b, test data were collected using an acceleration sensor mounted to the impactor and a drop tower impact system (Instron CEAST 9350). The preset impact energies were achieved by adjusting the drop height of the impactor and the compression degree of the spring. At least three specimens were tested for each energy level according to the ASTM D7136/D7136M-20 standard. Because the repeatability of the drop-weight impact test is acceptable, only one sample for impact energy value was used [31]. Finally, the nondestructive inspection characterizations were assessed by ultrasound C-scanning systems (Olympus NDT 2.10R25).

## 3. Finite Element Modeling

**Figure 3:**
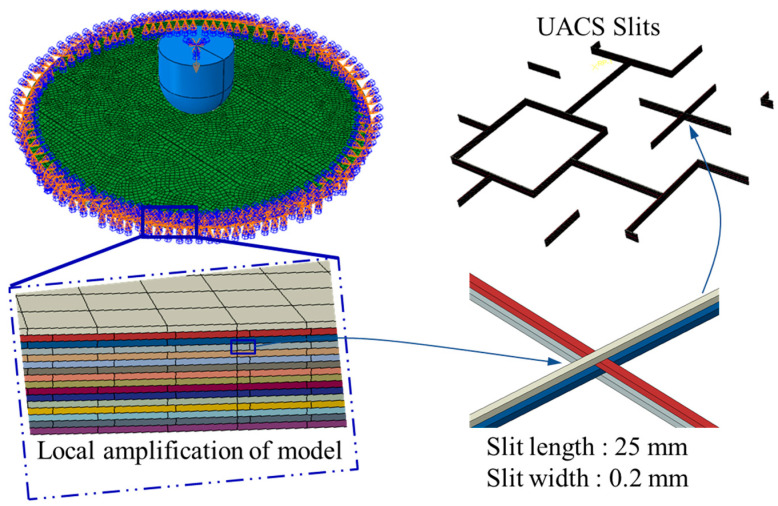
Model of UACS composite laminates under low-velocity impact.

In order to model the low-speed impact damage in UACS composite structures, a 3D finite element simulation model was created by using the ABAQUS/Explicit Dynamic simulation method. The researchers’ main priority was to analyze the impact damage problems of composite structures, such as the accurate experimental monitoring of the barely visible impact damage and the numerical simulation of impact response, for predicting the complex structural damage mechanisms in a fairly short time. The experiment was carried out at room temperature in an enclosed space; therefore, the numerical simulation did not consider the influence of ambient temperature. The 16 mm diameter hemispherical head impactor was simulated as an analytical rigid body. The model of the laminate was cut and given different material properties, as seen in Figure 3. The material of the slits in the model was defined as resin, and the other parts were defined as received CFRP prepreg [32]. All freedoms along the periphery of the plate were constrained to zero to simulate the experimental clamped conditions, and all freedoms of the indenter except the z-axis were constrained to zero to simulate the drop-weight low-velocity impact. The distance between the external surface of the impactor and the plate was set as 0 mm. The initial velocities of 1.23 m/s, 1.65 m/s and 2.04 m/s over the z-axis and 5.265 kg of mass were given to the indenter to generate three impact energies of 4, 7 and 11 J.

The eight-node solid elements with reduced integration (C3D8R) were used in the plate. Table 1 shows the details of the material properties of the prepreg used in this research. These parameters were provided by the manufacturer of prepreg (Weihai Guangwei) based on experimental analysis and empirical calculation. In addition, the zero-thickness eight-node three-dimension cohesive elements (COH3D8) were inserted between adjacent layers to simulate delamination. In order to improve the computational accuracy and efficiency, the mesh with an element size of 1 mm × 1 mm was used in the model. Therefore, the mesh of composite laminates consisted of 99216 C3D8R elements and 93015 COH3D8 elements. Regarding the interface cohesive element, Liu [33] selected the best parameters for the cohesive element in their work after comparing the impacts of various contact strengths on crack propagation damage. Table 2 lists the parameters of the interface cohesive elements that were employed in this investigation. All freedoms along the periphery of the plate were constrained to zero to simulate the experimental clamped conditions. A general contact algorithm in ABAQUS/Explicit was adopted to simulate contact between the impactor and the plate, as well as ply to ply in the laminate. The penalty method with a friction coefficient was used to describe the tangential behavior, while the hard contact method was employed to describe the normal behavior. Between the layers, there was an average friction coefficient of 0.5, and between the impactor and the plate, it was 0.3 [22,34]. Typically, the two categories used to describe the damage types of composite laminates under low-speed impact are intralaminar damage (matrix cracking and fiber breaking) and interlaminar damage. Damage simulations, including the damage initiation criterion and damage propagation law, can simulate the impact damage process. The equivalent displacement model selected in this paper has good accuracy for simulating damage development [22,30,35].

### 3.1. Johnson–Cook Elastic–Plastic Model

**Figure 4:**
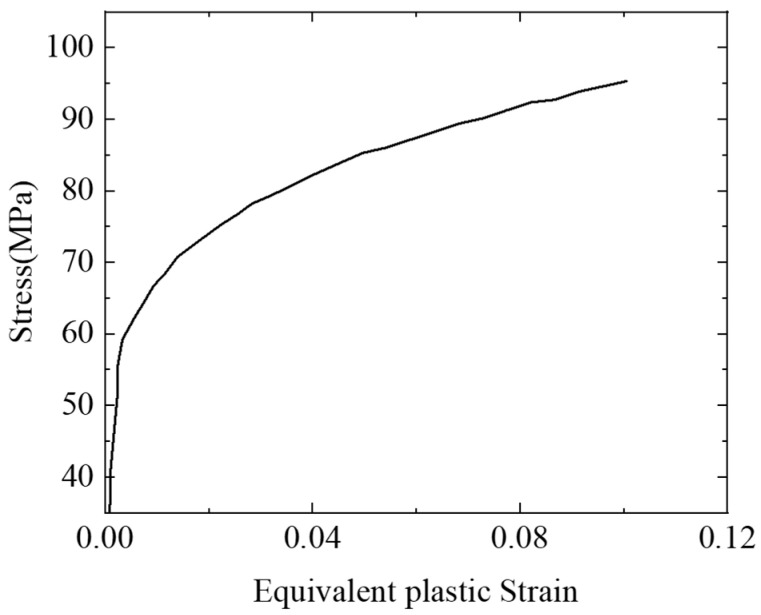
Stress-equivalent plastic strain curve of matrix resin [36].

The failure mechanism of slits filled with resin is a key factor associated with initial damage growth. The elastic–plastic phase of resin is mimicked by the Johnson–Cook model, and then, the ductile damage criterion determines the fracture to describe nonlinear stress–strain behavior and failure mechanism. In the present study, the Johnson–Cook plasticity model is expressed by the following Equation:

(1) $$\sigma^{0}=A+B{\left({\bar{\varepsilon }}^{\mathrm{pl}}\right)}^{n}$$

where ${\bar{\varepsilon }}^{\mathrm{pl}}$ is the equivalent plastic strain, and $\sigma^{0}$ is the static yield stress. A, B and n are material parameters. Referring to the curve fitting in a previous study [36], A = 35, B = 109.7 and n = 0.259 are applied for the failure analysis of resin, as shown in Figure 4.

### 3.2. Damage Initiation Criterion

One of the well-known standards for forecasting failure starts in composite structures is the Hashin criterion [37], which is constructed using formulas based on material strengths. The following are the four distinct Hashin criteria failure modes:

Fiber tensile failure: ($\sigma_{11}$ ≥ 0)

(2) $$F_{\mathrm{ft}}={\left(\frac{\sigma_{11}}{X_{T}}\right)}^{2}+{\left(\frac{\sigma_{12}}{S_{12}}\right)}^{2}+{\left(\frac{\sigma_{13}}{S_{13}}\right)}^{2}\geq 1$$

Fiber compression failure: ($\sigma_{11}$ < 0)

(3) $$F_{\mathrm{fc}}={\left(\frac{\sigma_{11}}{X_{C}}\right)}^{2}\geq 1$$

Matrix tensile failure: ($\sigma_{22}+\sigma_{33}$ ≥ 0)

(4) $$F_{\mathrm{mt}}={\left(\frac{\sigma_{22}+\sigma_{33}}{Y_{T}}\right)}^{2}+{\left(\frac{1}{S_{23}}\right)}^{2}\left(\sigma_{23}{}^{2}-\sigma_{22}{\;\sigma }_{33}\right)+{\left(\frac{\sigma_{12}}{S_{12}}\right)}^{2}+{\left(\frac{\sigma_{13}}{S_{13}}\right)}^{2}\geq 1$$

Matrix compression failure: ($\sigma_{22}+\sigma_{33}$ < 0)

(5) $$F_{\mathrm{mc}}={\left(\frac{\sigma_{22}+\sigma_{33}}{2S_{23}}\right)}^{2}+\frac{\sigma_{22}+\sigma_{33}}{Y_{C}}\left[{\left(\frac{Y_{C}}{2S_{23}}\right)}^{2}-1\right]+\frac{1}{S_{23}^{2}}\left(\sigma_{23}^{2}-\sigma_{22}\sigma_{33}\right)+{\left(\frac{\sigma_{12}}{S_{12}}\right)}^{2}+{\left(\frac{\sigma_{13}}{S_{13}}\right)}^{2}\geq 1$$

The fiber direction tensile and compressive strengths are represented by the variables $X_{T}\;$ and $X_{C}$ in the equations above; $S_{12}$, $S_{13}$ and $S_{23}$ are the corresponding shear strengths, while $Y_{T}$ and $Y_{C}$ represent the tensile and compression strengths in the cross-sectional direction.

### 3.3. Damage Evolution Law

Once the damage initiation standard is attained, a damage progression law is needed for the damage development. The progressive damage model needs to be encoded in the user-specified subroutine, which incorporates the Hashin criterion and a computation technique for equivalent movement in the damage propagation model. Here, a gradual deterioration approach coupled with a modified numerical model suggested by Zhou et al. [22] is utilized to describe the progression of intralaminar damage. Each failure mode’s damage variable is written as:

(6) $$G_{I}=\frac{1}{2}\sigma_{I,\mathrm{eq}}\varepsilon_{I,\mathrm{eq}}l_{c}$$

(7) $$d_{I}=\frac{\delta_{I,\mathrm{eq}}^{f}\left(\delta_{I,\mathrm{eq}}-\delta_{I,\mathrm{eq}}^{0}\right)}{\delta_{I,\mathrm{eq}}\left(\delta_{I,\mathrm{eq}}^{f}-\delta_{I,\mathrm{eq}}^{0}\right)}\;\left(d_{I}\epsilon [0,\;1],\;I=\mathrm{ft},\;\mathrm{fc},\;\mathrm{mt},\;\mathrm{mc}\right)$$

where $G_{I}$ is the fracture toughness of different failure modes; $l_{c}$ is the element’s characteristic length. The damage corresponding displacement of the related potential failure at initial and final failure is represented by $\delta_{I,\mathrm{eq}}^{f}$ and $\delta_{I,\mathrm{eq}}^{0}$, respectively.

The constitutive Equation with nine independent cross-ply-layered composite laminates treated as orthorhombic anisotropy materials was adopted to appreciate the mechanical response. The degraded flexibility matrix $S_{d}$ and the corresponding degraded stiffness matrix $C_{d}$, added with different damage variables in the constitutive Equations, are expressed as:

(8) $$S_{d}=\left[\begin{array}{cccccc} \frac{1}{d_{f}E_{11}} & -\frac{\nu_{21}}{E_{22}} & -\frac{\nu_{31}}{E_{33}} & & & \\ -\frac{\nu_{12}}{E_{11}} & \frac{1}{d_{m}E_{22}} & -\frac{\nu_{32}}{E_{33}} & & & \\ -\frac{\nu_{13}}{E_{11}} & -\frac{\nu_{23}}{E_{22}} & \frac{1}{E_{33}} & & & \\ & & & \frac{1}{d_{f}d_{m}G_{12}} & & \\ & & & & \frac{1}{d_{f}d_{m}G_{23}} & \\ & & & & & \frac{1}{d_{f}d_{m}G_{31}} \end{array}\right]$$

(9) $$C=\frac{1}{\Delta }\left[\begin{array}{cccccc} d_{f}E_{11}\left(1-d_{m}\nu_{23}\nu_{32}\right) & d_{f}d_{m}E_{11}\left(\nu_{21}+\nu_{23}\nu_{31}\right) & d_{f}E_{11}\left(\nu_{31}+d_{m}\nu_{21}\nu_{32}\right) & & & \\ & d_{m}E_{22}\left(1-d_{f}\nu_{13}\nu_{31}\right) & d_{m}E_{22}\left(\nu_{32}+d_{f}\nu_{12}\nu_{31}\right) & & & \\ & & E_{33}\left(1-d_{f}d_{m}\nu_{12}\nu_{21}\right) & & & \\ & & & {\Delta d}_{f}d_{m}G_{12\;} & & \\ & & & & {\Delta d}_{f}d_{m}G_{23} & \\ & & & & & {\Delta d}_{f}d_{m}G_{13} \end{array}\right]$$

(10) $$\begin{array}{c} d_{f}=\left(1-d_{\mathrm{ft}}\right)\left(1-d_{\mathrm{fc}}\right)\\ d_{m}=\left(1-S_{\mathrm{mt}}d_{\mathrm{mt}}\right)\left(1-S_{\mathrm{mc}}d_{\mathrm{mc}}\right)\\ \Delta =1-d_{f}d_{m}\nu_{12}\nu_{21}-d_{m}\nu_{23}\nu_{32}-d_{f}\nu_{13}\nu_{31}-2d_{f}d_{m}\nu_{21}\nu_{32}\nu_{13} \end{array}$$

where $d_{I}$ (I=ft, fc, mt, mc) are the fiber and matrix damage variables computed using the damage evolution model. $S_{\mathrm{mt}}$ and $S_{\mathrm{mc}}$ are newly introduced coefficients that are adjusted to 0.96 in the current study in order to manage the shear stiffness loss [33,38].

### 3.4. Interlaminar Damage

Before damage initiation and evolution, the traction–separation model is in the elasticity phase. Interlaminar delamination occurs with the initiation of damage simulated by the quadratic nominal stress criterion, and corresponding reduction behavior is determined by the BK fracture criterion [39]. Equations (11) and (12) express the cohesive zone model used to predict the growth of delamination damage (12).

(11) $$\;{\left\{\frac{\langle t_{n}\rangle }{t_{n}^{o}}\right\}}^{2}+{\left\{\frac{t_{s}}{t_{s}^{o}}\right\}}^{2}+{\left\{\frac{t_{t}}{t_{t}^{o}}\right\}}^{2}=1$$

(12) $$G^{C}=G_{n}^{C}+\left(G_{s}^{C}-G_{n}^{C}\right){\left\{\frac{G_{S}}{G_{T}}\right\}}^{\eta }\left(G_{S}=G_{s}+G_{t},{\;G}_{T}=G_{n}+G_{S}\right)\;$$

where $G_{n}$, $G_{s}$ and $G_{t}$ are normal and shear directions fracture energies, and $t_{n},\;t_{s}$, and $t_{t}$ stand for normal and shear tractions; $t_{n}^{o}$, $t_{s}^{o}$, and $t_{t}^{o}$ stand for interface normal and shear strength. The quantities $G_{n}^{C}$, $G_{s}^{C}$ and $G^{C}$ are critical fracture energy of the normal, the shear and total directions, respectively, and η, set as 1.45, is a material parameter.

## 4. Results and Discussion

As part of the impact test process at various energies (4, 7 and 11 J), the force–time, force–displacement and energy–time curves were documented. For numerical simulation, the analysis was carried out from damage modes, including fiber tensile damage, intralaminar matrix cracking and fatigue cracks at the interfaces.

### 4.1. Impact Response

**Figure 5:**
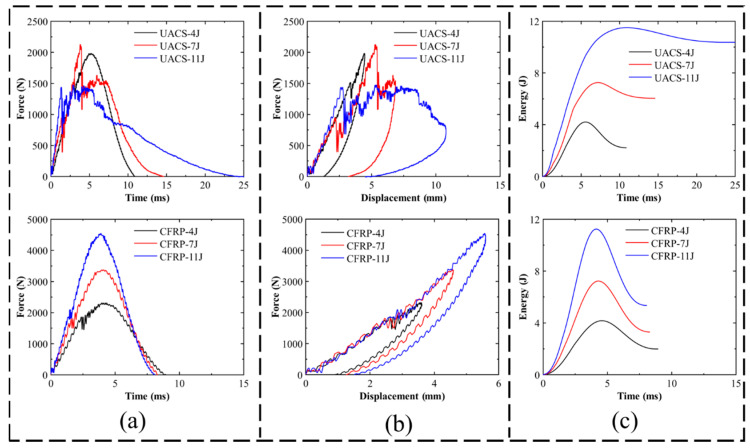
Comparison of experimental result of CFRP and UACS laminates under 4 J, 7 J and 11 J impact energies. (**a**) Force–time curve, (**b**) force–displacement curve and (**c**) energy–time curve.

Energy–time, force–displacement and force–time histories of both continuous CFRP and UACS laminates were compared for each impact energy in order to make an accurate comparison of the impact performance of the experimental data (see Figure 5). The energy–time curve of UACS laminates associated with penetration at 11 J will be discussed in the comparison of the numerical and experimental results. For both continuous CFRP and UACS laminates, 11 J impact energy presents the highest impact time, displacement and energy, whereas laminates impacted with 4 J display similar impact response behaviors due to no obvious damage occurring in the laminates under this impact energy. Table 3 lists the final results from the experiment with various impact energies. The peak force of UACS and CFRP laminates differs by 14% at 4 J, and subsequently, with the failure of UACS laminates, the increasing gap indicates a decrease in load capacity for UACS laminates. However, in the case of non-failure (4 J), the UACS laminates show better toughness with a maximum displacement of 4.48 mm compared to 3.61 mm of CFRP. Compared to the continuous CFRP laminates, the UACS laminate shows a larger maximum displacement by about 24%, and its peak force is about 37% less than continuous CFRP laminates under the impact energy of 7 J due to the fiber breakage of UACS laminates. The final energy absorbed by the CFRP laminates is about 1.99 J for the impact energy of 4 J, which is approximately 10% lower than that of the UACS (2.21 J). For each of the three types of impact energy, the impact energy–time histories are displayed in Figure 5c. For 11 J impact energy, the ultimate energy absorbed by the laminates in the event of damage and friction is 10.38 J and 5.34 J for UACS and CFRP, respectively. In the case of 7 J, however, the UACS value of 6.05 J is closer to the CFRP value of 3.29 J. It appears that absorbed energy increases with increasing impact energy, and it is clear that the UACS laminates absorb more energy than the CFRP laminates.

**Figure 6:**
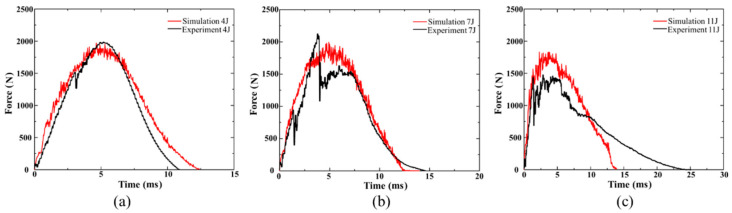
Comparison of numerical and experimental impact force–time curves of UACS laminates under impacts at (**a**) 4 J, (**b**) 7 J and (**c**) 11 J.

**Figure 7:**
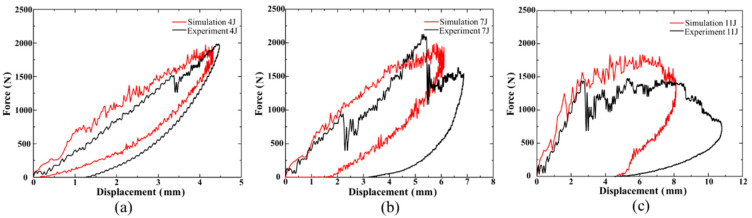
Comparison of numerical and experimental force–displacement curve of UACS laminates under impacts (**a**) 4 J, (**b**) 7 J and (**c**) 11 J.

Figure 6 exhibits the correlation between experimental and numerical impact force–time graphs of UACS laminates at three impact energies. Figure 6 and Figure 7 show that the initial contact stage of the impact force–time curves always exhibits substantial oscillation, which can be attributed to the early fracture of the layers on the impact side. Meanwhile, sudden drops in the force and displacement under the energy of 7 J occur at 2 ms and 3.95 ms, respectively. In the beginning, the delamination grows quickly at the slits, which causes the initial drop in force. With damage accumulation due to the continuous absorption of kinetic energy by the laminate, the failure of the laminate leads to a steeper drop than the first drop. When the initial kinetic energy increases to 11 J, the drop in force is obviously earlier. The establishment of material structure in the numerical model is based on the ideal situation compared to the experiments. Therefore, the accurate simulation of the mechanical response of brittleness composite laminates is relatively difficult. Premature failure of impact side layers causes the penetration of composite laminates, and that is why the impact process of 11 J is prolonged. As a result of the actions of laminate vibration, damage initiation and progression with various modes, the impact force increases swiftly and fluctuates dynamically. As the value gets closer to the maximum, extremely intense oscillations occur, and the rate at which the impact force increases declines. The impact force then starts to decrease, indicating the impactor will bounce after that. In contrast to the experiment test’s limited acquisition points, a large amount of data sets may be obtained in brief time intervals, and simulation can effectively depict the intensive oscillation patterns of the impact force. The simulation’s rapid oscillation history depicts the laminates’ increasing damage due to several failure types. It does, however, differ somewhat from the actual experimental method. The impact force–displacement graphs for the three impact energies are shown in Figure 7. Due to the crack formation of laminates throughout the experiment, the maximum displacement speculated by the numerical model was consistently a little less than the results of the experiment. Figure 7a–c again demonstrate that the maximum displacement of the impactor increases as the impact energy does. Due to the bending deformation of composite laminates, the displacement of the indenter exceeds the thickness of the laminates by a significant margin.

**Figure 8:**
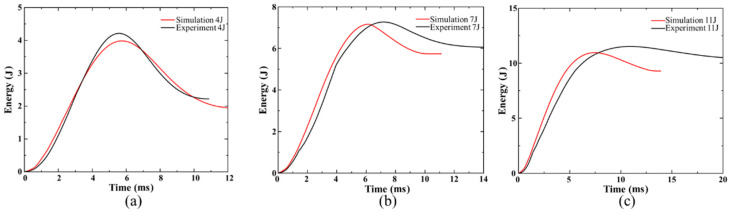
Comparison of numerical and experimental energy–time curves of UACS laminates under impacts (**a**) 4 J, (**b**) 7 J and (**c**) 11 J.

The impact energy–time graphs for three impact energies are depicted in Figure 8. After contact, the impactor’s kinetic energy quickly starts to transfer to the composite laminates. The elastic energy of the laminates absorbs a portion of the kinetic energy, while the damage and vibration of the laminates disperse the remainder. An accurate numerical model is used to simulate the peak energy absorption. As shown in Figure 8b,c, the energy absorbed by laminates is released by means of fiber brittle fracture and delamination near the slits, and the indenter takes longer in the experimental test than in the numerical simulation to reach zero velocity and stable bouncing back velocity; as a result, the last absorbed energy determined by the numerical methods is never more than the results of the experiment. The laminates absorb more energy as the impact energy rises, indicating that the damage issue worsens.

### 4.2. Post Impact C-Scan Inspection

**Figure 9:**
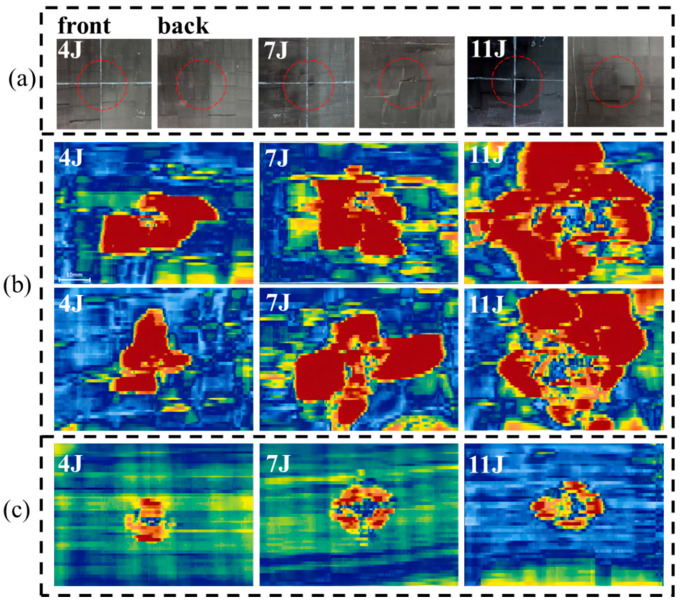
Comparison of C-scan maps and damaged specimens impacted at 4 J, 7 J and 11 J. (**a**) Damaged UACS laminates, (**b**) C-scan maps of UACS laminates and (**c**) C-scan maps of continuous CFRP laminates.

Additional information on the damage mechanisms is provided by the C-scan maps of UACS and continuous CFRP laminates (see Figure 9). The impact-damaged specimens are also compared to the C-scan maps. Each damaged UACS laminate is represented by either the hemispherical impactor (front side) or a fracture surface (back side) corresponding to the 4 J, 7 J and 11 J impact energies. The 4 J impact does not cause significant damage to the specimens, whereas the 11 J impact induces the maximum damage in comparison to the other energies. At 7 J, the hemispherical indentation on the impacted side becomes clear (this is the first energy that makes an indentation over the BVID in the specimens). Different from the hemispherical indentation, the delamination area of the UACS laminates is shaped like a square influenced by slits. Additionally, there is a generalization of fiber splitting and major development of warp/weft direction cracks on the non-impacted side. Damage evolution in UACS is more severe than that of CFRP laminates. Obviously, for the highest impact energy in UACS, the delaminated areas reach about 20% of the specimen, as listed in Table 3. It can be concluded that the UACS laminates tend to delaminate to a greater extent than CFRP laminates at increasing impact energy.

### 4.3. Failure Modes Analysis

Matrix cracking, delamination and fiber breaking are the primary damage mechanisms for composite structures in low-velocity impacts. Even with little impact energy, matrix cracking and delamination can easily form, but fiber breaking only happens with considerably large impact energy. These damage types can sometimes be invisible from the outside of the laminates, yet they nonetheless significantly affect the remaining mechanical performance of composite structures. High tension between laminas and shear stresses in the impact zone result in delamination, which is an essential component of impact damage.

#### 4.3.1. Fiber Tensile Damage

In the initial stage of the impact process, the resin in the slits is the first to crack after the laminate is impacted. Then, the slits are completely fractured to release energy, which is one of the reasons why UACS laminates absorb more energy. Because the slit direction of adjacent layers is vertical, the fibers are subjected to a large amount of shear force when the slits are damaged, resulting in lower impact performance of the laminate. However, in this case, the interlaminar delamination induced by the notch failure will limit the damage extending from the impact center point to the outside.

**Figure 10:**
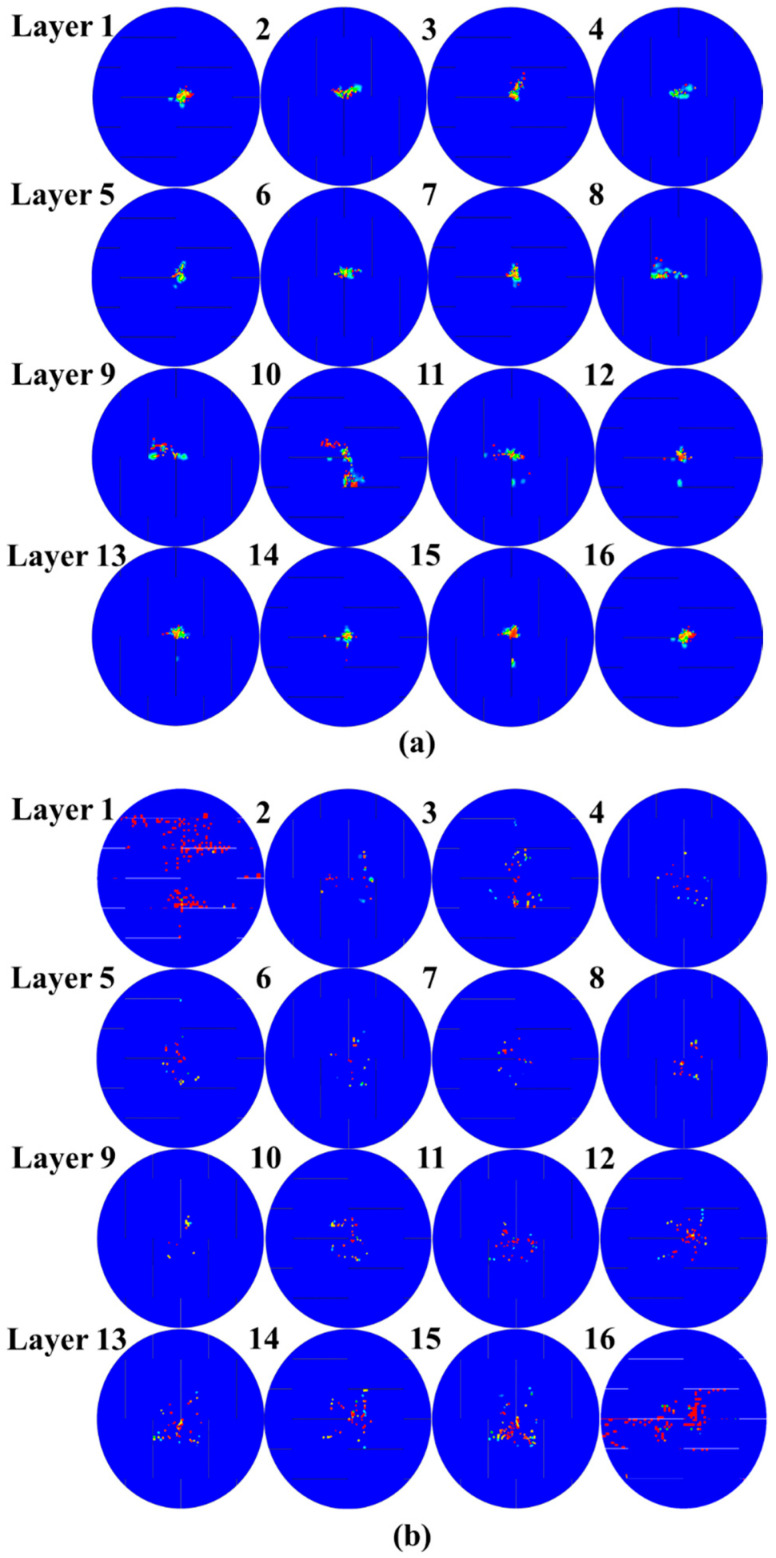

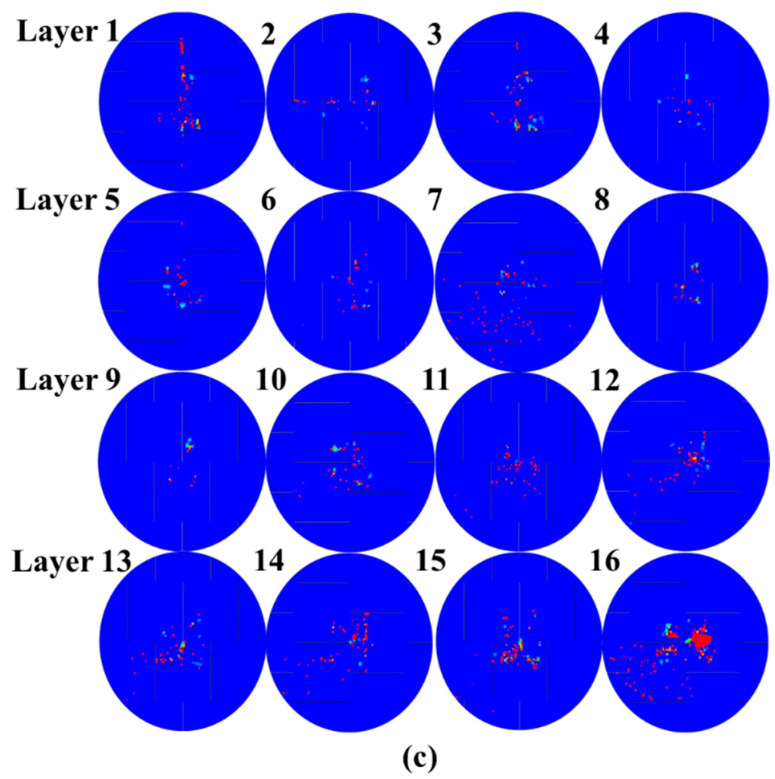
Fiber tensile damage of UACS laminates under impacts at (**a**) 4 J, (**b**) 7 J and (**c**) 11 J.

As shown in Figure 10a,b, slight damage appears in the central region at 4 J impact energy; as a comparison, large-area fiber breakages are induced with the path of damage evolution perpendicular to the slits under 7 J impact energy, which will result in the penetration of UACS laminate at higher energy. Figure 10c illustrates fiber tensile damage before penetration at 12.8 ms of the impact process. The layers on the impact and back side present similar imaging with 4 J; namely, damage accumulates primitively in the center zone because this region bears impact load in the first stage and then expands along the fiber direction subjected to shear loading.

#### 4.3.2. Matrix Tensile Damage and Compression Damage

**Figure 11:**
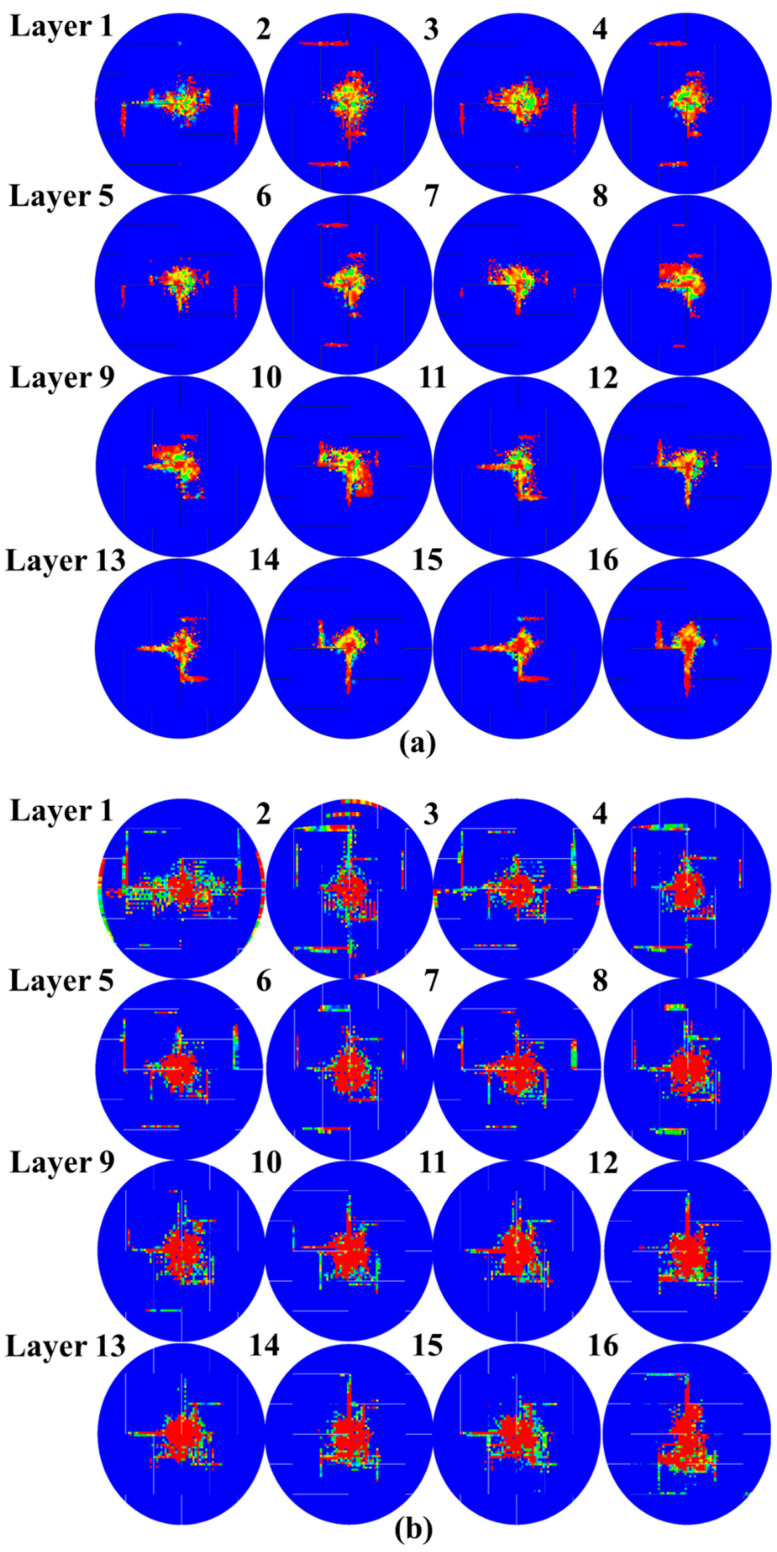

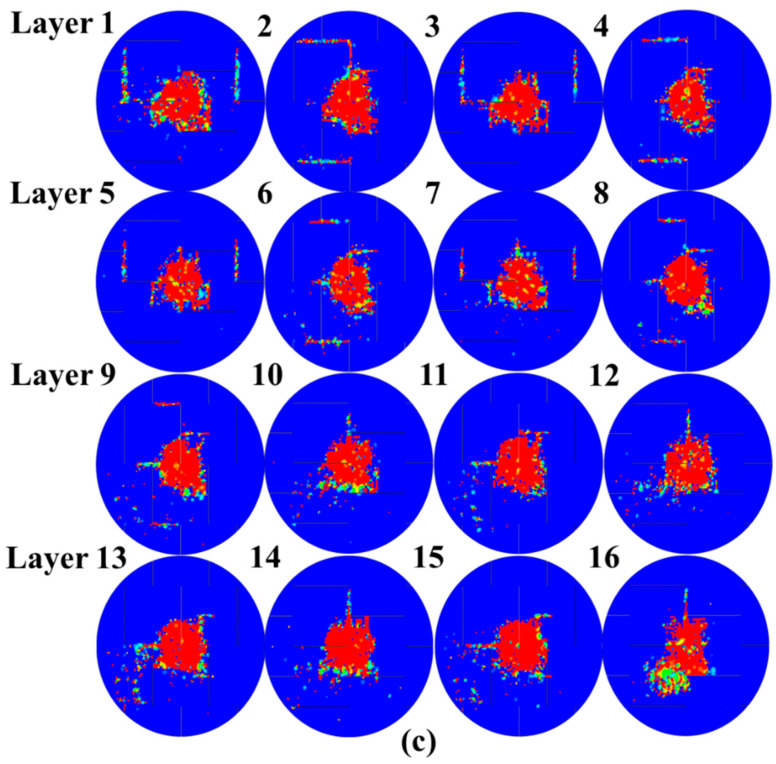
Matrix tensile damage of UACS laminates under impacts at (**a**) 4 J, (**b**) 7 J and (**c**) 11 J.

**Figure 12:**
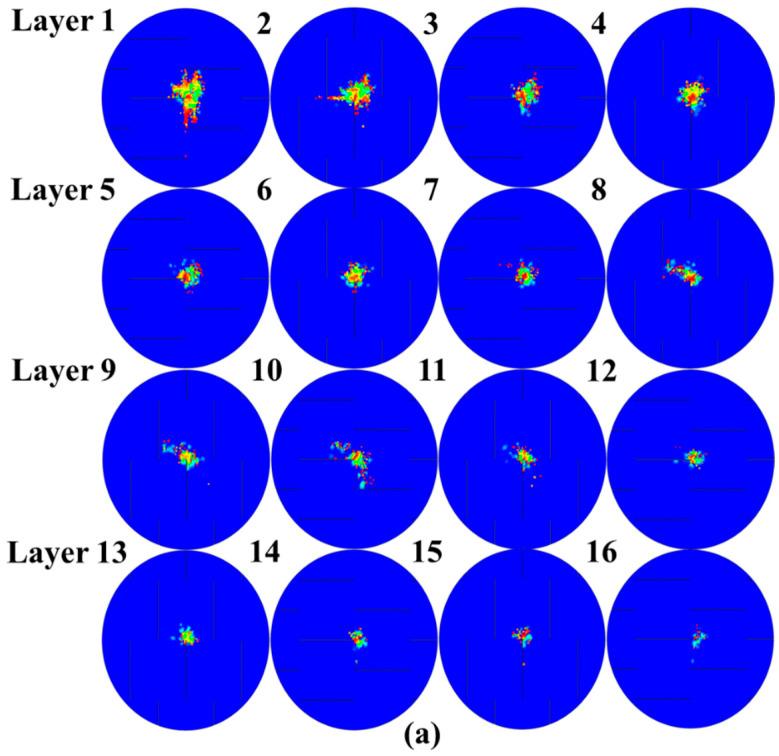

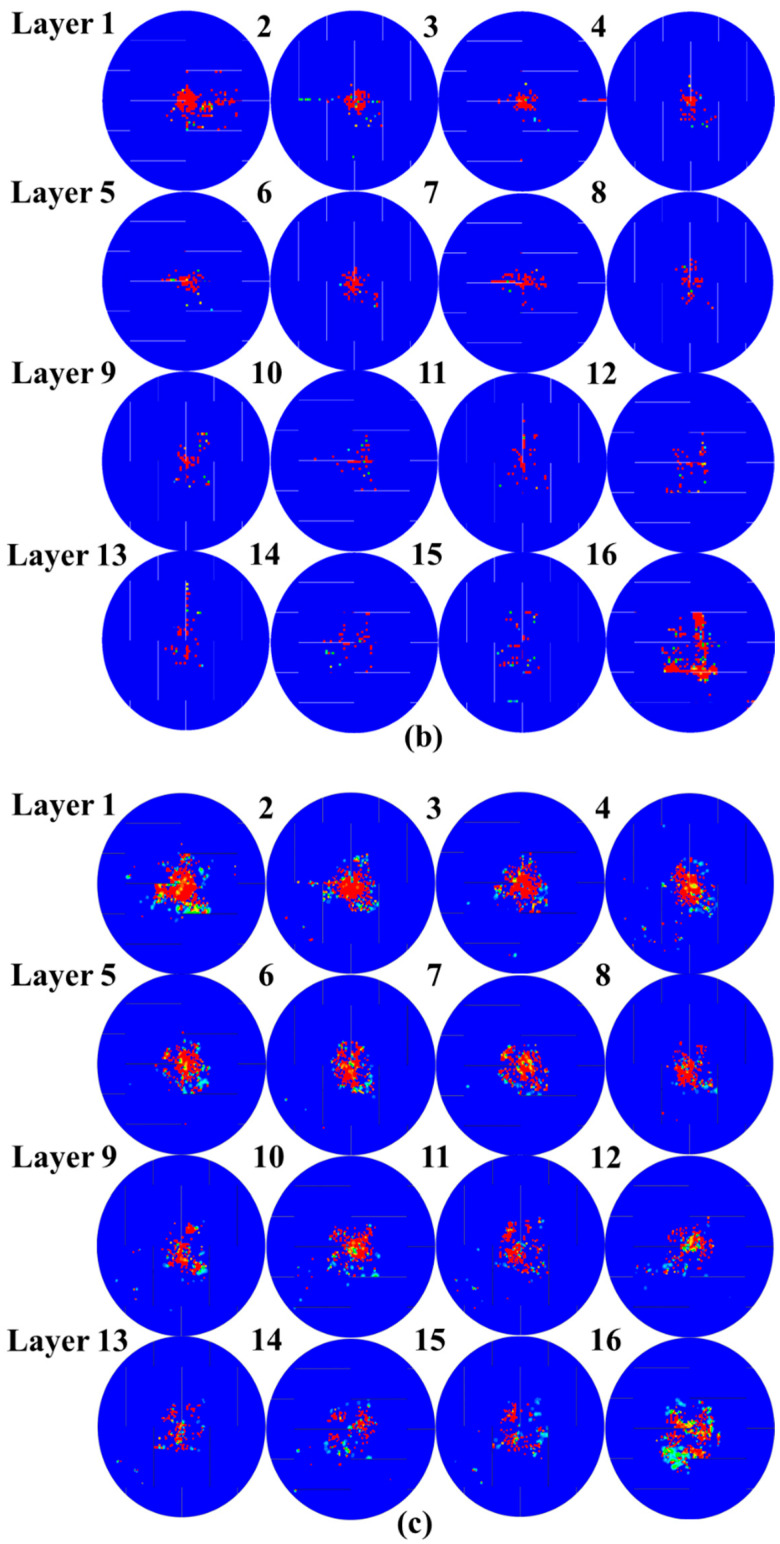
Matrix compression damage of UACS laminates under impacts at (**a**) 4 J, (**b**) 7 J and (**c**) 11 J.

Figure 11 depicts how matrix tensile damage is distributed across each layer of the numerical models. The damage is evidently concentrated near the place of impact. Moreover, it is clear that when the equivalent layer is farther away from the contact side, the anticipated matrix tensile damage area is bigger. This can be defined by the way the laminates deform and fail, showing how matrix tensile damage begins upon the last laminate and then spreads toward the top layers. It is noteworthy that the addition of slits changes the damage propagation rule, especially in Figure 11a,b, where the matrix damage offers the shape of a “vertical bar” in the vicinity of the slits as the threshold of the damage area observed by ultrasound C-scanning systems. Similarly, from Figure 11c, which shows the damage condition before the failure of the laminate, it can be seen that the matrix damage of back side layers expands from the central region to damaged slits. The predictions of matrix compression damage within every ply are shown in Figure 12. To be more specific, the damage region for every ply from the 7 J impact is greater than the damage region from the 4 J impact. According to this research, matrix compression damage starts on the contact side and spreads toward the backside, which is why the top four layers of the matrix show the most damage. For the majority of layers, the matrix compression damage region is less than the previously analyzed matrix tensile damage region. Given the direction of the fiber, matrix compression damage tends to concentrate at the site of impact and rarely extends in other directions.

#### 4.3.3. Delamination of UACS Composite

**Figure 13:**
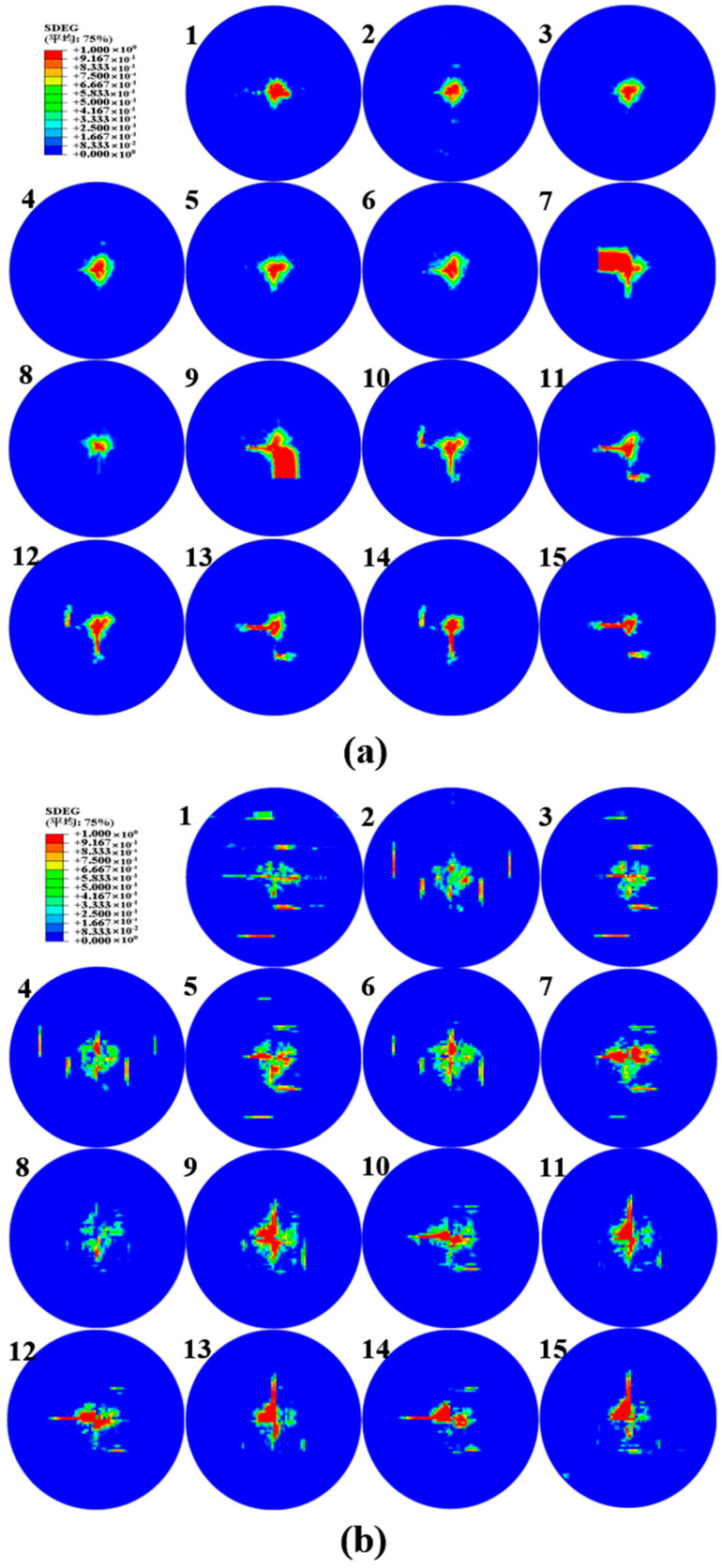

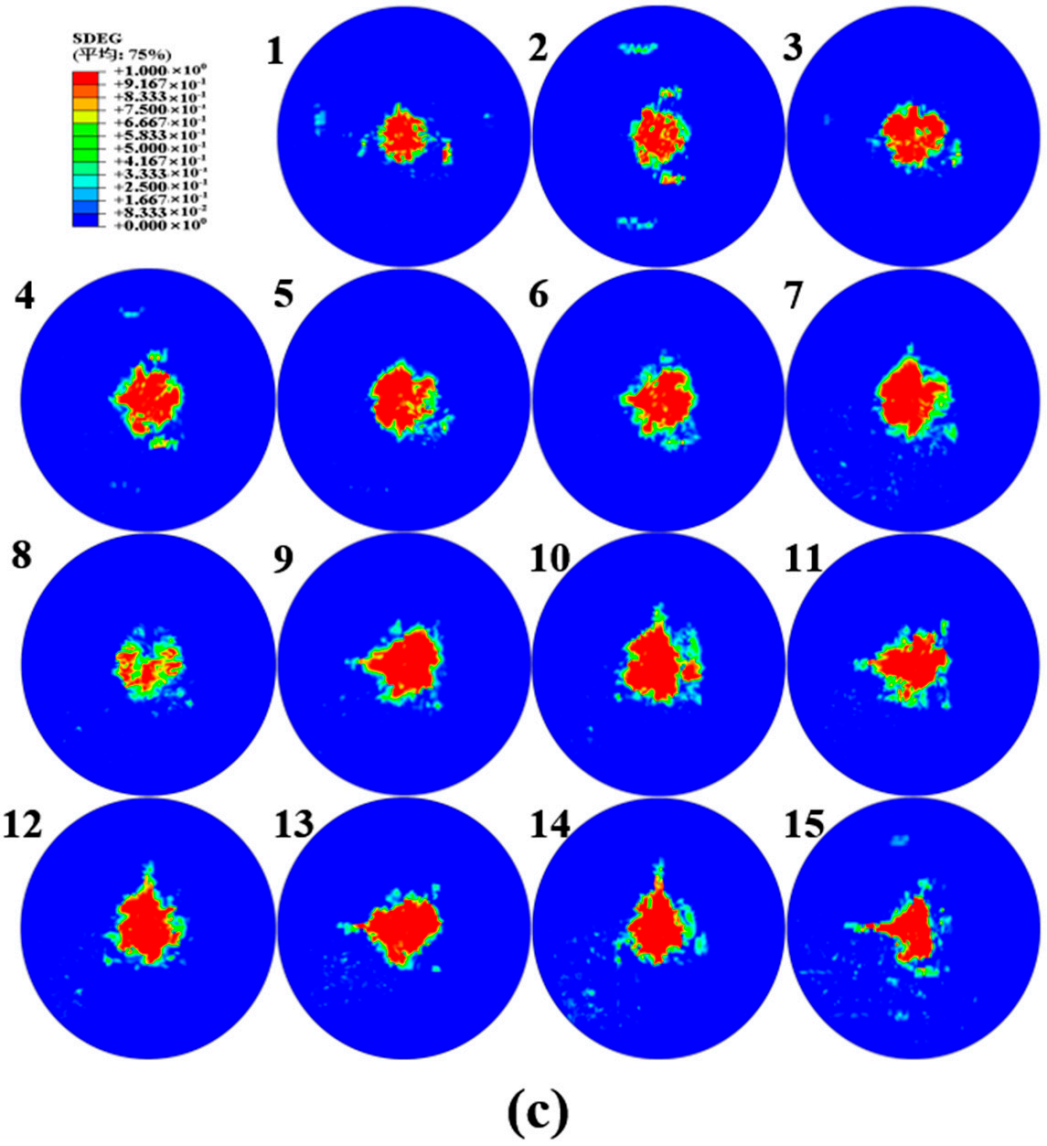
Delamination area of the UACS composite laminates under impacts at (**a**) 4 J, (**b**) 7 J and (**c**) 11 J.

For each interlaminar interface on the impact region, Figure 13 depicts the delamination morphology. For the overall value of the scalar damage variable (SDEG), variables above that indicate whether the BK fracture criterion is satisfied or not, a value that is less than 1.0 indicates that the criterion is not satisfied, while a value of 1.0 or higher indicates that the delamination occurred. In total, the UACS cross-ply composite laminates examined here have fifteen interfaces. As can be seen under the 4 J energy level, the majority of the delamination region in each interface is not entirely damaged but only partly fails. The composite structures could unexpectedly fail as a result of this partly failed delamination continuing to spread secretly during subsequent use. The largest critical delamination can be observed at the 11 J energy level followed by 7 J.

It is common knowledge that the stress concentration in CFRP laminates responding to low-velocity impacts is only high when close to the impact area and appears to reduce along the laminates’ in-plane and thickness directions. However, for the UACS laminates, the existence of slits makes the delamination generate along the slit direction of the upper layer. This special delamination area appears at UACS laminates under different energy levels, especially at 7 J. As shown in Figure 13b, the delamination area consists of two parts, the elliptical impact region loading by hemispherical punch and the slits delamination. Unlike the delamination area under the impact energy of 7 J, the slits delamination, as shown in Figure 13a, only occurs on several backside layers. With the rise in impact energy, the delamination area propagates from central to slits, as shown in Figure 13c, resulting in the penetration of UACS laminates under the impact energy of 11 J.

## 5. Conclusions

This work examined the dynamic mechanical behavior and damage progression of UACS laminates with vertical slits using a series of low-velocity impact tests and a PDM-based finite element simulation integrating intralaminar damage and delamination. From the numerical and experimental results, the following can be drawn:

1. At the same impact energy level, the UACS laminates with vertical slits show lower peak force than the continuous CFRP laminates. The continuous CFRP laminate shows a limited damage area, whereas the delamination of the UACS laminate offers the shape of a “vertical bar” in the vicinity of the slits, resulting in a larger damage area. With the increase in impact energy, the maximum displacement of UACS laminates is larger than that of continuous CFRP laminates and shows superior energy absorption capability.
2. The finite element model is developed by the Johnson–Cook elastic–plastic model and PDM, and good accordance is determined between the numerical simulation and experimental measurements on the peak force of the laminates, as well as the maximum displacement and the energy absorbed during the impact event. Moreover, the effect of the dimension of slits, the stacking sequence and the thickness of laminates on the low-velocity impact performance of UACS laminates could be investigated further in future work.

## Figures and Tables

**Table 1:** Material properties of continuous CFRP laminate.

Parameters	Value
Density	1600 kg/m^3^
Stiffness properties	$E_{11}\;$ = 145 Gpa, $E_{22}$ = $E_{33}$ = 9 Gpa, $G_{12}$ = $G_{13}$ = 4.4 Gpa, $G_{23}$ = 3.7 Gpa, $\nu_{12}$ = $\nu_{13}$ = 0.3, $\nu_{23}$ = 0.4
Strength properties	$X_{T}$ = 2600 Mpa, $X_{C}$ = 1050 Mpa, $Y_{T}$ = 62 Mpa, $Y_{C}$ = 192 Mpa, $S_{12}$ = $S_{13}$ = 90 Mpa, $S_{23}$ = 52 MPa
Fracture Energy	$G_{\mathrm{ft}}$ = 82 N/mm, $G_{\mathrm{fc}}$ = 70 N/mm, $G_{\mathrm{mt}}$ = 0.22 N/mm, $G_{\mathrm{mc}}$ = 1.1 N/mm

**Table 2:** Material properties of interface cohesive elements.

Parameters	Value
Elastic modulus	$E_{n}$ = $E_{s}$ = $E_{t}$ = 5 GPa/mm
Strength properties	N = S = T = 30 MPa
Fracture energy	$G_{n}^{c}$ = 0.32 N/mm; $G_{s}^{c}$ = 1 N/mm
Relevant coefficient η	1.45

**Table 3:** Experimental values of different specimens.

Specimen	Impact Energy (J)	Peak Force (kN)	Maximum Displacement (mm)	Absorbed Energy (J)	Damage Area (mm^2^)
CFRP	4	2.33 ± 0.08	3.51 ± 0.18	1.89 ± 0.03	153 ± 14.50
	7	3.35 ± 0.11	4.49 ± 0.23	3.19 ± 0.13	250.5 ± 25.25
	11	4.56 ± 0.21	5.53 ± 0.46	5.24 ± 0.08	311 ± 22.05
UACS	4	1.97 ± 0.09	4.18 ± 0.30	2.31 ± 0.08	485.75 ± 41.75
	7	2.13 ± 0.15	6.86 ± 0.58	6.05 ± 0.38	889.75 ± 56.05
	11	1.46 ± 0.11	10.79 ± 0.71	10.38 ± 0.24	1230 ± 87.85

## Data Availability

Supporting data is available on request.

## Abbreviations

**Nomenclature**		$G_{\mathrm{mt}}$	Fracture toughness for tensile damage of matrix
		$G_{\mathrm{fc}}$	Fracture toughness for compressive damage of fiber
Symbol	definition	$G_{\mathrm{ft}}$	Fracture toughness for tensile damage of fiber
$\sigma_{11}$	Component stress acting at fiber direction	$F_{\mathrm{ft}}$	Hashin criteria for fiber failure in tension
$\sigma_{22}$	Component stress acting at normal to the fiber	$F_{\mathrm{fc}}$	Hashin criteria for fiber failure in compression
$\sigma_{33}$	Component stress acting at normal to the ply plane	$F_{\mathrm{mt}}$	Hashin criteria for matrix failure in tension
$\sigma_{12}$	Shear stress acting at the ply plane	$F_{\mathrm{mc}}$	Hashin criteria for matrix failure in compression
$\sigma_{13}$	Shear stress acting transverse to ply plane	$l_{c}$	Element characteristic length
$\sigma_{23}$	Shear stress acting transverse to ply plane	$d_{\mathrm{ft}}$	Damage variable for fiber failure in tension
$\sigma^{0}$	Static yield stress	$d_{\mathrm{fc}}$	Damage variable for fiber failure in compression
$E_{11}$	Young modulus at fiber direction	$d_{\mathrm{mt}}$	Damage variable for matrix failure in tension
$E_{22}$	Young modulus at normal to fiber	$d_{\mathrm{mc}}$	Damage variable for matrix failure in compression
$E_{33}$	Young modulus at normal to the ply plane	η	Material parameter of the BK fracture criterion
$G_{12}$	Shear modulus acting at the ply plane	$S_{\mathrm{mc}}$	Coefficients to manage the shear stiffness
$G_{13}$	Shear modulus acting at the ply plane	$S_{\mathrm{mt}}$	Coefficients to manage the shear stiffness
$G_{23}$	Shear modulus acting transverse to ply plane	$\delta_{I,\mathrm{eq}}^{f}$	Damage corresponding displacement of failure at initial failure
$\nu_{12}$	Poisson’s ratio acting at the ply plane	$\delta_{I,\mathrm{eq}}^{0}$	Damage corresponding displacement of failure at final failure
$\nu_{13}$	Poisson’s ratio acting at the ply plane	$E_{n}$	Elastic modulus of normal direction
$\nu_{23}$	Poisson’s ratio acting transverse to ply plane	$E_{s}$	Elastic modulus of shear direction
$X_{T}$	Fiber direction tensile strength	$E_{t}$	Elastic modulus of shear direction
$X_{C}$	Fiber direction compressive strength	$t_{n}^{o}$	Interface normal strength
$Y_{T}$	Tensile strength in the cross sectional direction	$t_{s}^{o}$	Interface shear strength
$Y_{C}$	Compressive strength in the cross sectional direction	$t_{t}^{o}$	Interface shear strength
$S_{12}$	Shear strength acting at the ply plane	$G_{n}^{c}$	Critical fracture energy of the normal direction
$S_{13}$	Shear strength acting at the ply plane	$G_{s}^{c}$	Critical fracture energy of the shear direction
$S_{23}$	Shear strength acting transverse to ply plane	$G^{C}$	Critical fracture energy of the total direction
$G_{\mathrm{mc}}$	Fracture toughness for compressive damage of matrix	${\bar{\varepsilon }}^{\mathrm{pl}}$	Equivalent plastic strain
